# High rate copper and energy recovery in microbial fuel cells

**DOI:** 10.3389/fmicb.2015.00527

**Published:** 2015-06-19

**Authors:** Pau Rodenas Motos, Annemiek ter Heijne, Renata van der Weijden, Michel Saakes, Cees J. N. Buisman, Tom H. J. A. Sleutels

**Affiliations:** ^1^Wetsus, European Centre of Excellence for Sustainable Water TechnologyLeeuwarden, Netherlands; ^2^Sub-department of Environmental Technology, Wageningen UniversityWageningen, Netherlands

**Keywords:** microbial fuel cells, bioelectrochemical systems, metal recovery, copper

## Abstract

Bioelectrochemical systems (BESs) are a novel, promising technology for the recovery of metals. The prerequisite for upscaling from laboratory to industrial size is that high current and high power densities can be produced. In this study we report the recovery of copper from a copper sulfate stream (2 g L^-1^ Cu^2+^) using a laboratory scale BES at high rate. To achieve this, we used a novel cell configuration to reduce the internal voltage losses of the system. At the anode, electroactive microorganisms produce electrons at the surface of an electrode, which generates a stable cell voltage of 485 mV when combined with a cathode where copper is reduced. In this system, a maximum current density of 23 A m^-2^ in combination with a power density of 5.5 W m^-2^ was produced. XRD analysis confirmed 99% purity in copper of copper deposited onto cathode surface. Analysis of voltage losses showed that at the highest current, most voltage losses occurred at the cathode, and membrane, while anode losses had the lowest contribution to the total voltage loss. These results encourage further development of BESs for bioelectrochemical metal recovery.

## Introduction

Heavy metals pose a serious problem when they are released into the environment due to their toxicity for humans and its negative effect on biodiversity (Norgate and Rankin, 2002). Therefore, removal, and recovery of heavy metals, remediation of polluted sites, and decontamination of waste streams is needed in heavy metal and mining industries. Today, the conventional method to recover metals, such as copper, is by electrowinning, which uses electric power to electrochemically reduce dissolved metals to their metallic form. As metals are becoming more scarce and expensive (Watkins and McAleer, 2006; Geman and Smith, 2013), the revenues compensate for the electricity costs. However, considerable amounts of electrical energy are still required in electrolysis cells (2.23 kW h kg^-1^ of Copper; Vegliò et al., 2003). In comparison, bioelectrochemical systems (BESs) can provide the energy by the bioelectrochemical degradation of organic compounds present in waste waters, for instance, acetate (electron donor), at a so called bioanode. By coupling this bioanode to a metal reducing cathode, waste water treatment can be coupled to the recovery of heavy metals in a clean and sustainable way. When additional energy is harvested from these two reactions, the system is known as a microbial fuel cell (MFC). It is also possible to add extra electrical energy to drive and accelerate a reduction reaction at the cathode, in that case, the system is known as a microbial electrolysis cell (MEC) (Logan et al., 2008). The anode and the cathode compartment are generally separated by a membrane to simultaneously avoid mixing of the electrolytes and in theory, these membranes allow ions to pass selectively, so charge neutrality is maintained.

ter Heijne et al. (2010) showed the proof of principle of this concept where they combined bioelectrochemical oxidation of acetate with the reduction of copper. In their system, electricity was produced while copper was removed and plated onto a solid electrode. A maximum current density of 3.2 A m^-2^ was achieved in a setup with a flat plate graphite electrodes and a bipolar membrane (BPM). Since then, other studies have reported MFCs in which copper was reduced at the cathode, reaching current densities ranging from 0.9 to 7 A m^-2^ with, for example, an increased surface area of the anode compared to the cathode (Modin et al., 2012; Zhang et al., 2012).

To make BESs practical and economically suitable to replace existing technologies, higher current densities are required. To reach high current densities, it is essential to reduce the voltage losses by reducing the internal resistance. Sleutels et al. (2012) analyzed the effect of the internal resistance on the practical applicability of BES. A resistance below 40 mΩ m^2^ was considered acceptable for BES applications to achieve sufficiently high current and power densities to make BESs economic. Besides economic studies based on internal resistance, other attempts have been done to estimate feasibility of BESs using life-cycle assessment (LCA) analysis (Foley et al., 2010; Pant et al., 2011; Sleutels et al., 2012).

On the other hand, copper recovery is a highly valuable application for BES research. The recovery of valuable products allows for higher internal resistances compared to the recovery of energy alone (Sleutels et al., 2012). Copper recovery as a cathode reaction can potentially lead to high current densities due to its low overpotential. It is therefore, a suitable cathode reaction to demonstrate that MFCs can be operated at high current densities.

The main objective of this study was to increase the current and power density of a copper reducing MFC. Our approach was to reduce the internal resistance compared to previous studies by four changes: (i) decreasing the distance between electrodes, (ii) use an anion exchange membrane (AEM), (iii) use a copper plate as cathode, and (iv) use carbon felt as anode material.

First, the distance between anode and cathode was reduced from 3 to 0.5 cm (ter Heijne et al., 2010). Second, an AEM is known to have lower internal resistance than other membranes when applied in MEC (Rozendal et al., 2007; Sleutels et al., 2009a). As the BPM resulted in high energy losses; the internal resistance was expected to be reduced by using an AEM. Third, cathode material was changed from graphite paper to copper, which is a material conventionally used in copper electroplating. Fourth, anode material was replaced from graphite paper to carbon felt to achieve high specific surface area for electroactive biofilm growth. To make this anode material available for microbial activity, the solution was forced via a perpendicular flow through this felt as shown by Sleutels et al. (2009b, 2011).

We operated this new cell design at different current densities and analyzed its performance in terms of power production and voltage losses.

## Materials and Methods

### Research Set Up

**FIGURE 1:**
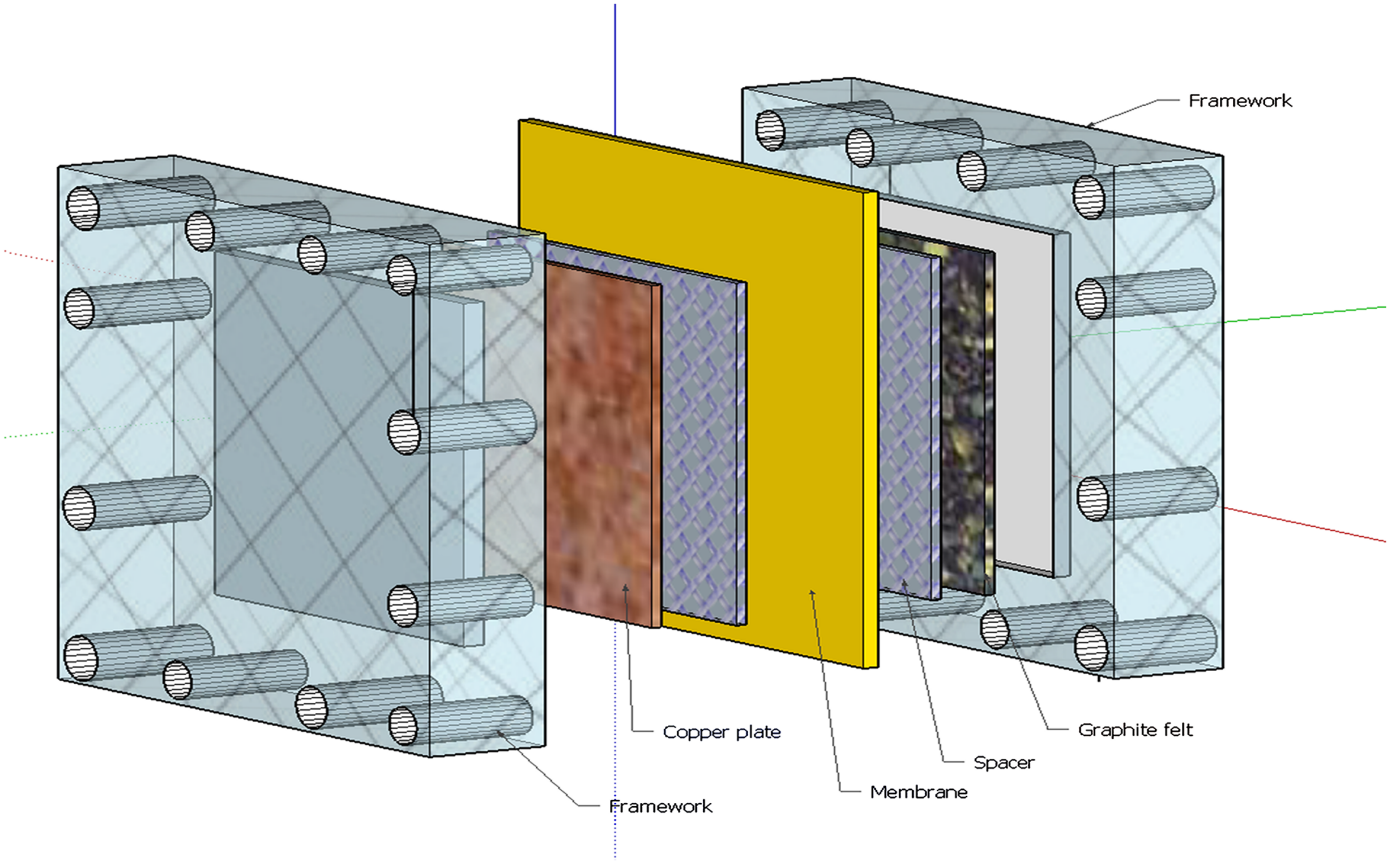
**Cell design 3D view with copper electrode (brown), membrane (yellow), spacer (clear blue patterned), graphite felt (black with white dots), and framework (transparent)**.

The cell used in these experiments was similar to the one used by Kuntke et al. (2014). This cell was built using 10 cm × 10 cm metallic copper plates as cathode and 10 cm × 10 cm carbon felt of 1.5 mm thickness as anode. A Ralex^®^AEM (MEGA a.s., Stráž pod Ralskem, Czech Republic) was placed in between the anode and cathode compartment. To force the electrolyte to flow perpendicular through the electrodes, 1.2 mm spacer (64% open; PETEX 07-4000/64, Sefar BV, Goor, Netherlands) was placed between the membrane and the electrodes in both compartments (**Figure 1**). The anolyte was forced to flow toward the membrane through the electrode (Sleutels et al., 2011). The anode, cathode, spacer material, and membrane were kept in place by bolting them in between two PMMA endplates.

The anode compartment was controlled at 30°C via a water flow through the outer wall in the anode recirculation bottle. Only the cathode temperature was measured and it oscillated between 20 and 23°C. Nitrogen was flushed continuously through anolyte and catholyte to keep anaerobic conditions in anode and cathode compartments. Anode and cathode potentials were measured against Ag/AgCl 3 M reference electrode (ProSense, Oosterhout, Netherlands; 201 mV vs. Standard Hydrogen Electrode). Voltage losses across the membrane were measured as the voltage difference between the two reference electrodes placed in catholyte and anolyte. Cell voltage and potentials of cathode, anode, and membrane were continuously recorded together with pH and temperature in both compartments using a data logger (Endress+Hauser Messtechnik GmbH+Co., Rhein, Germany).

### Start-Up and Operation

At the beginning of the experiment a 2 g L^-1^ Cu^2+^ solution was prepared dissolving CuSO_4_ 5⋅H_2_O as catholyte. The anode influent (2mL min^-1^) consisted of synthetic waste water solution containing 20 mM acetate, phosphate buffer (10 mM, pH 7), nutrients, and vitamins as described in ter Heijne et al. (2008). The anode was fed with an excess of acetate to make sure the current production was not limited by substrate availability. Acetate was measured over time to ensure an excess of electron donor in the anode to produce current. Any competing processes at the anode for example methanogens were taken into consideration. Anolyte and catholyte had a constant recirculation rate of 200 ml min^-1^. The total catholyte volume was 10 L. Cathode and anode pH were controlled at pH 3 and pH 7, respectively, with two dose pumps (STEPDOS 08, KNF, Germany) with 0.1 M H_2_SO_4_ and 0.1 M KOH. No supporting electrolyte was used. The conductivity of the solution was not measured during the experiment, only at the beginning of the experiment, and the values are 9.2 mS/cm for the catholyte and 6.2 mS/cm for the anolyte. The presence of an excess of acetate and sulfate (transported from the cathode) in the anolyte resulted in sulfate reduction (due to sulfate reducing bacteria; Hamilton, 1985) and consequently, in a consumption of protons. Hence, acid addition was required in the anode compartment. It should be noted that addition of chemicals to control the pH is not a sustainable solution for practical applications but it was used here to be able to study the maximum performance of the system for copper removal. The cell started up at a constant resistance of 1 kΩ. The resistor was switched to a lower value (stepwise using 1 kΩ, 500 Ω, 250 Ω, and 100 Ω) after the anode potential had stabilized at a value of -450 mV vs Ag/AgCl. During this growing period of 2 weeks stable conditions were ensured using buffer solution as catholyte for oxygen reduction and measuring stable voltages over the entire cell. During the measurements that are reported in the results section below, the resistances were lowered sequentially from 1000 Ω to 0.5 Ω and catholyte was changed to copper sulfate solution. For each resistance steady state values over a period of 24 h were used to calculate the polarization curve.

### Analyses

Copper concentration was measured using inductively coupled plasma optical emission spectrometry (ICP-OES) Perkin Elmer Optima 5300 (Perkin Elmer, Groningen, Netherlands). The consumed acetate during the experiment was tracked every 24 h measuring TOC (Total Organic Carbon) and IC (Inorganic Carbon) that was correlated with the concentration of acetate and bicarbonate using Shimadzu TOC-L CPH combustion TOC analyzer (Shimadzu Benelux, ’s-Hertogenbosch, Netherlands). The gas composition in the headspace of the cells was also examined with a gas chromatographer [Varian Inc. (Part A) – CP-4900 Micro-GC].

The deposited copper on the electrode was scratched with a knife from the surface, weighed in a test tube and dissolved in 5 ml of 33% HNO_3_. Then, 5 ml of Mili-Q water was added to dilute the solution. Then the solution obtained was diluted 1000 times again and measured by ICP–OES Perkin Elmer Optima 5300 to determine the amount of copper plated on the electrode.

### Calculation

The theoretical cell voltage (*E*_emf_ in V) can be calculated from the Gibbs free energy change of the overall reaction occurring at the cathode and anode, while the practical value can be directly measured at open cell conditions

(1) $$E_{emf}=\frac{-\Delta G_{r}}{nF}$$

Where, ΔG_r_ is the Gibbs free energy of the overall reaction (kJ mol^-1^), *n* is the number of electrons transferred in the reaction, *F* is Faraday constant (96485 C mol^-1^).

The contribution of the partial voltage losses to the total voltage loss inside the cell can be calculated according to

(2) $$E_{CELL}=E_{emf}-\eta_{Cath}-\eta_{Anod}-\eta_{mem}$$

Where *E*_CELL_ is the voltage measured during cell operation (V), η_Cath_ is the measured cathode overpotential (theoretical cathode potential-measured cathode potential; V), η_Anod_is the measured anode overpotential (theoretical anode potential-measured anode potential; V), and η_mem_ is the voltage lost across the membrane (cathode ref potential-anode ref potential; V).

## Results and Discussion

### Current and Power Output

**FIGURE 2:**
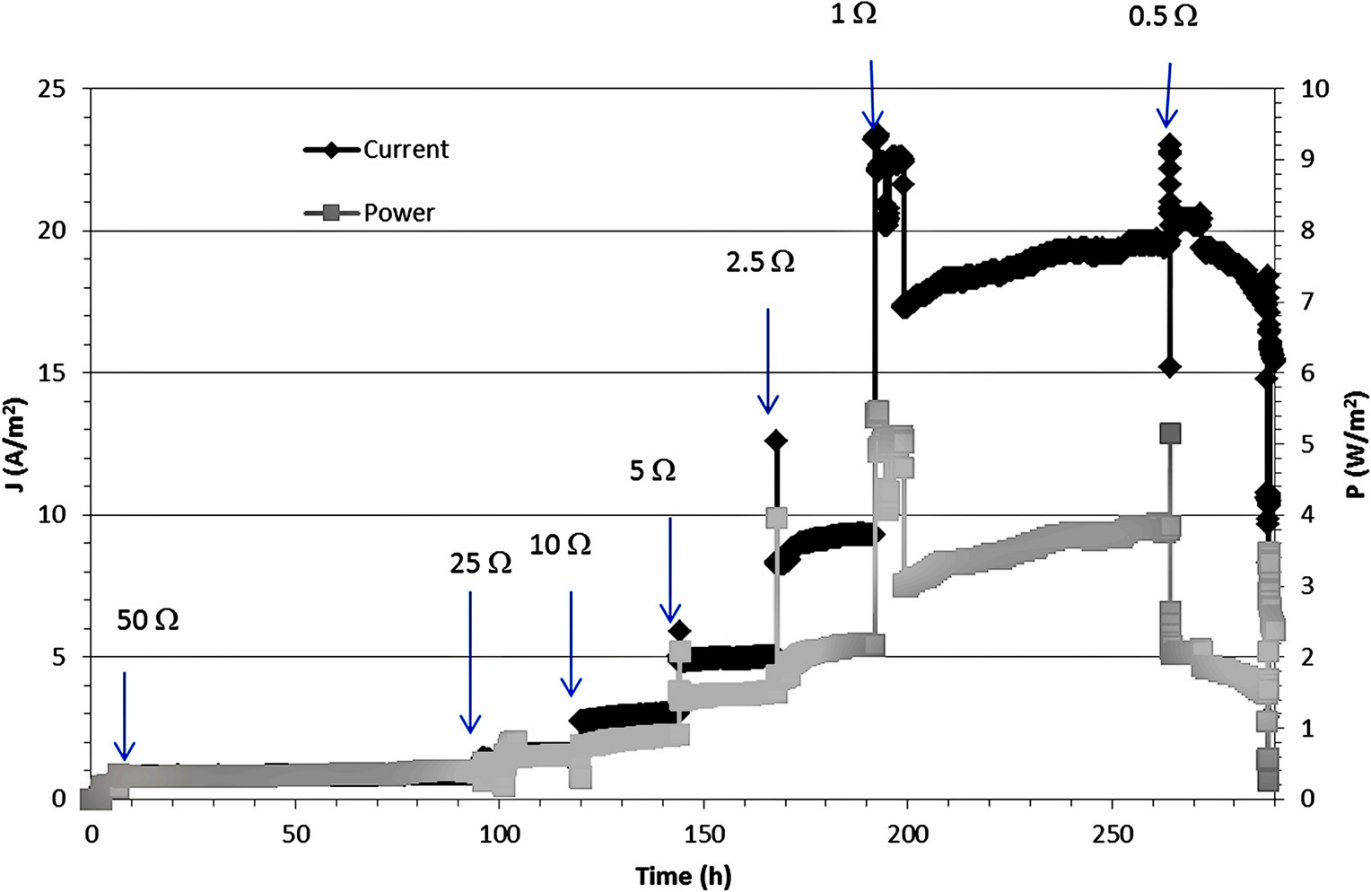
**Evolution of current density (black) and the power density (grey) shown vs. time during the entire range of the experiment.** The arrows indicate when resistances were changed.

**FIGURE 3:**
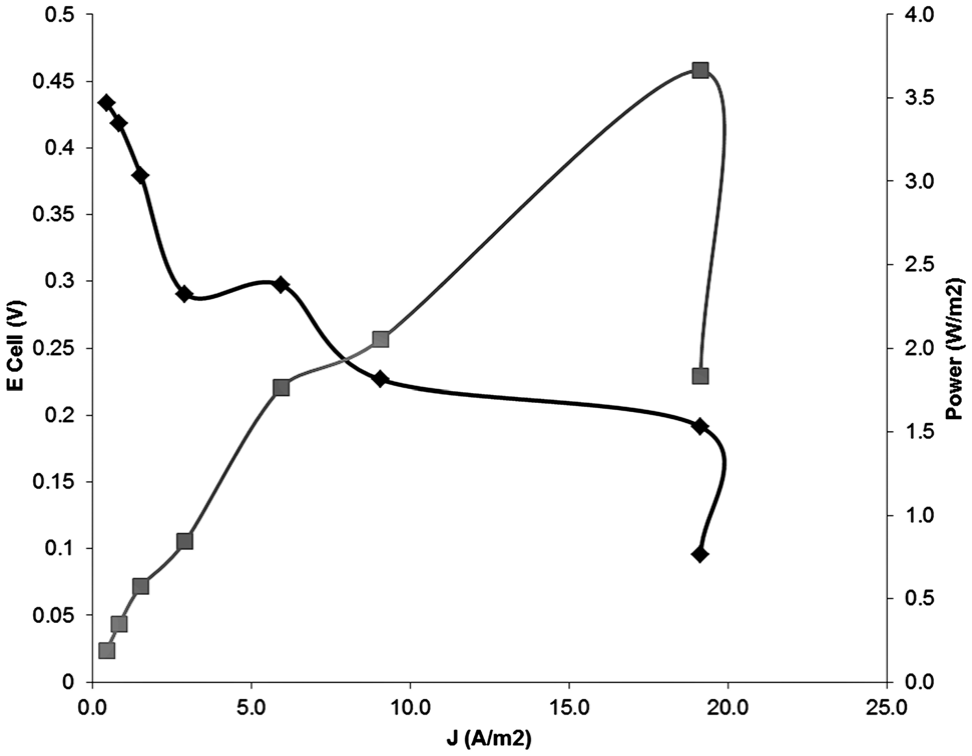
**Polarization curve of the copper reducing microbial fuel cell (MFC).** The potential is represented vs. current density (black) and the power is shown versus current density (grey).

**Figure 2** shows the current and power density generated over the entire experimental period. The arrows indicate the moment at which the value of the external resistance was lowered. The current density increased from 0.5 A m^-2^ at an external resistance of 100 Ω up to a stable value of 19 A m^-2^ at an external resistance of 1 Ω. The peak current density was 23 A m^-2^ and at this maximum current density, the peak power density was 5.5 W m^-2^. When the resistor was further decreased to 0.5 Ω, the current density did not increase further, and the power output dropped from 4.0 to 2.0 W m^-2^. **Figure 3** shows the polarization curve of the MFC, summarizing the whole experimental period, using the average values for cell voltage, current, and power density at each external resistor. The open circuit voltage (OCV) measured the first day was 485 mV, which is lower than the theoretical value of 575 mV.

During the whole period only 60% of the total amount of acetate was consumed by the biofilm, from this amount only the 26% was used to produce electricity. The detection of methane and sulfide in the head space of the recirculation bottle let us conclude that acetate was used for methane production and sulfate reduction by methanogens and sulfate reducers competing for the substrate with the electrogenic bacteria. The oxidation of acetate coupled to the reduction of sulfate by sulfate reducing bacteria was described by Muyzer and Stams (2008) via:

(3) $${\mathrm{Acetate}}^{-}{+SO}_{4}^{2-}\to {2HCO}_{3}^{-}{+HS}^{-}$$

### Analysis of the Performance

**FIGURE 4:**
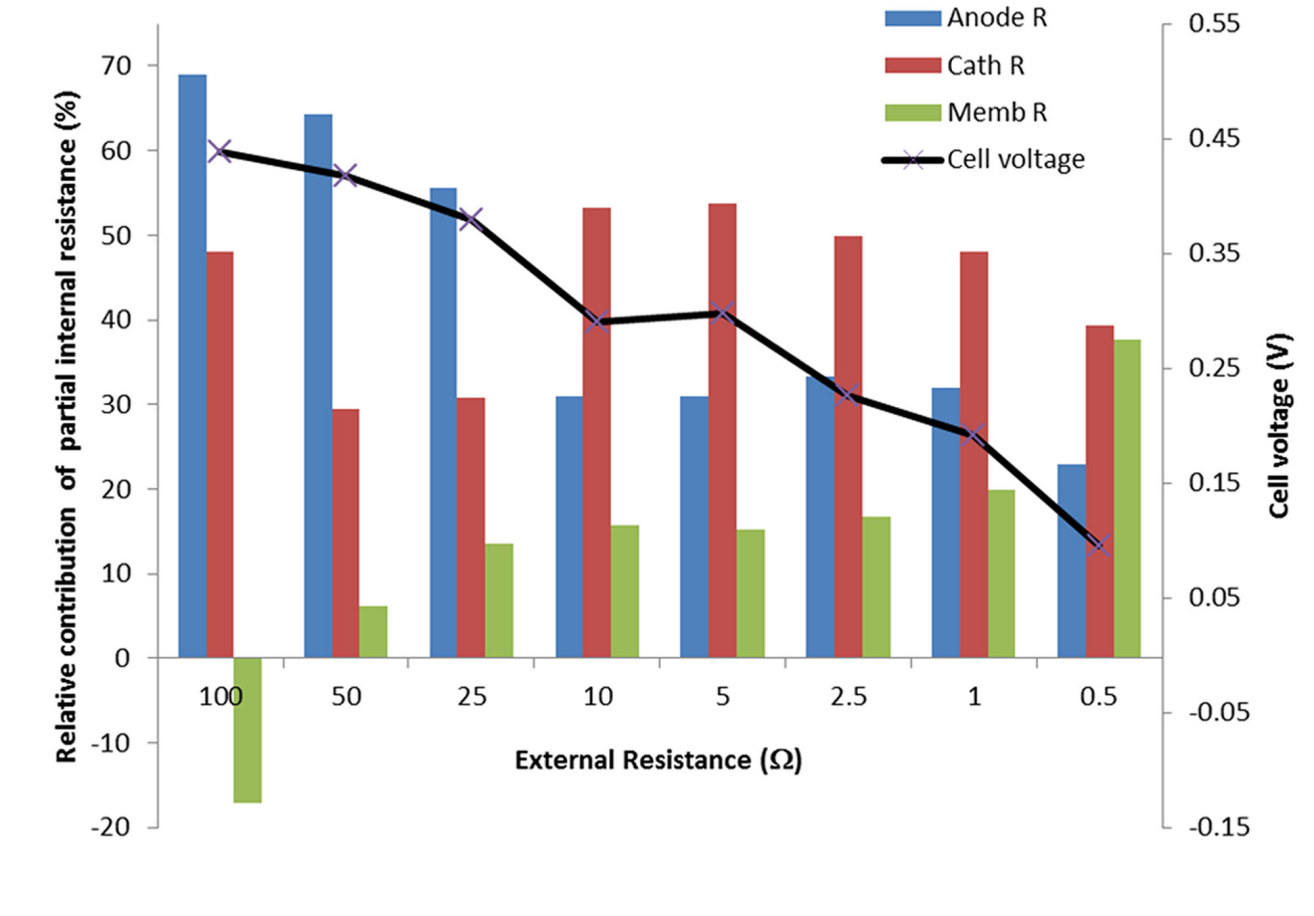
**Contribution of the cell components (anode, cathode, and membrane) to the total voltage loss in the system at different external resistors.** Also the cell voltage is shown for each external resistor.

To analyze the reason for the drop in power production in more detail, we have analyzed the separate contributions of anode, cathode, and voltage losses across the membrane throughout the experimental period in line with a previous study from ter Heijne et al. (2006). **Figure 4** shows the relative contribution of the voltage losses for these three main components of the system: at every external resistance. The voltage loss over the membrane was measured using the reference electrode inserted in the anode and cathode compartment. Then, the ionic resistance of the electrolyte (anode and cathode) is measured as part of the membrane voltage loss. Note that **Figure 4** shows the contribution of the three components, the three of them summing up to 100%. This was done to illustrate the differences with time more clearly. Obviously, at a high external resistance the produced current was lowest while at a decreasing external resistor the produced current increased (**Figure 2**). So overall, the internal resistance of the system decreased with time.

With an increase in current density, the contribution of anode overpotential to the total voltage losses decreased considerably from 68 to 23%. We observed an increase in anode voltage from -450 to -400 mV vs Ag/AgCl for the first steps with high external resistances. However, when current reached its maximum values, the anode potential raised only to -350 mV vs Ag/AgCl. Apparently the biofilm was not affected by the high protons production rate that is associated with the current production. Instead an increase in the voltage loss over the membrane was observed. At increasing current densities voltage losses across the membrane increased from -50 mV on first day to 180 mV on ninth day. This value of -50 mV was result of the difference in chemical potential between anolyte and catholyte due to the difference in composition of these electrolytes. Sulfate was transported through the membrane from catholyte to anolyte. This chemical potential contributes positively to the cell potential. At the highest current reached, the dominant losses occurred at the cathode, approximately around 50% of total losses contribution between 10 Ω external resistance and 1 Ω external resistance. The increase in current density at lower external resistance was also reflected in an increase of the cathode overpotential. Cathode potential went down from 50 mV during first day to -60 mV vs Ag/AgCl at the end of the experiment due to the decrease in Copper concentration.

**FIGURE 5:**
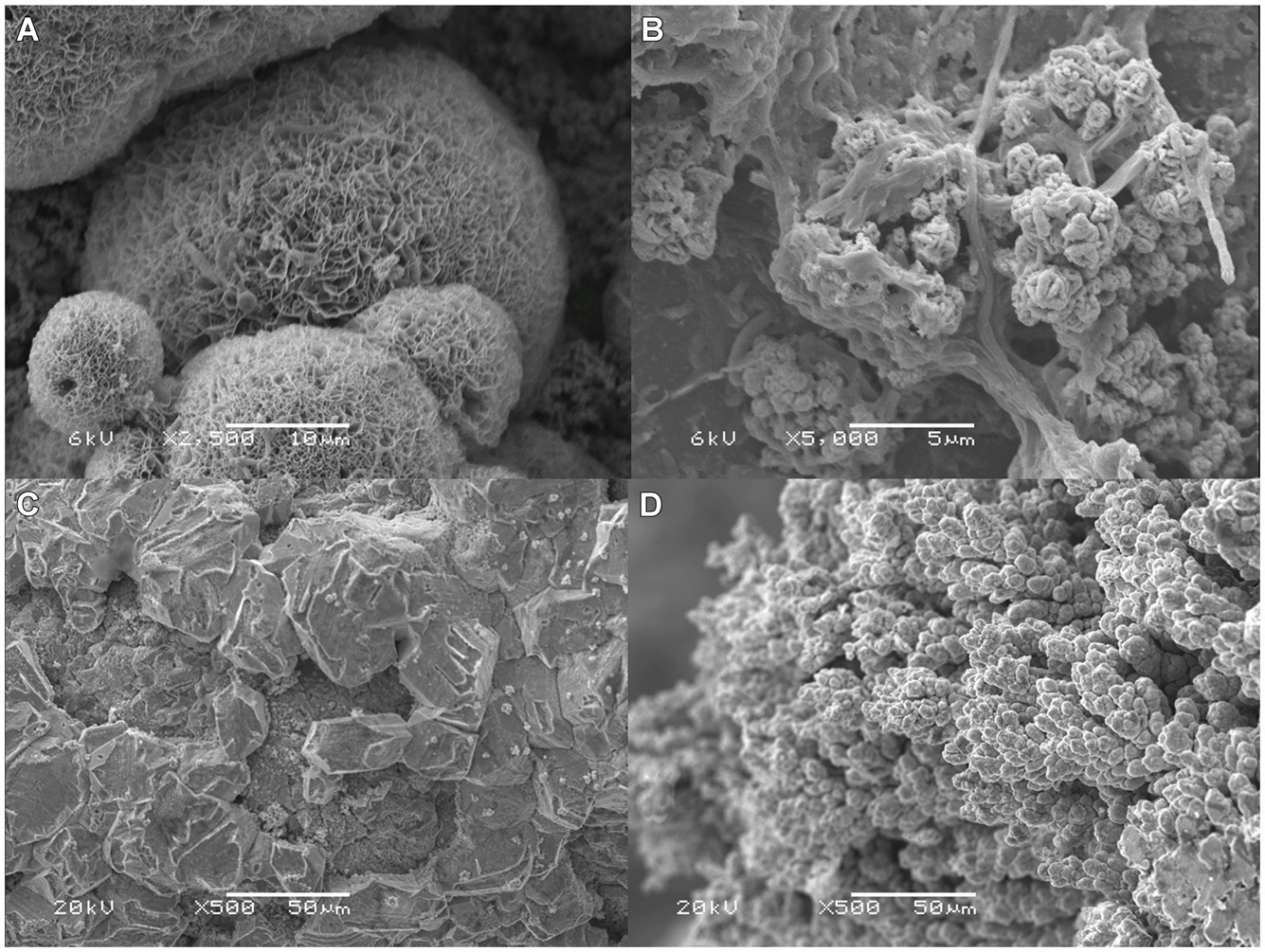
**(A)** SEM image of crystalline scaling from cathode side on anion exchange membrane (AEM), **(B)** SEM image of biofilm on anode side of AEM. **(C)** SEM image of smooth copper deposited on flat copper surface **(D)** SEM image of the dendrites formed on cathode surface.

An increase in the membrane voltage losses is likely at higher current densities since more ions need to be transported in the same time period. Additionally, the increase in membrane voltage losses can also be related to scaling. At the end of the experiment, when the cell was disassembled, precipitates were found in the catholyte and on the membrane surface. **Figures 5A,B** show the scanning electron microscope (SEM) image of scaling and biofouling in the cathode and in the anode side respectively on top of the membrane. Analysis of these structures with electron diffraction X-ray (EDX) shows the presence of copper, sulfur, and phosphor, which lead us to believe that the precipitates are copper sulfates, copper sulfides, and copper phosphates. Scaling is more likely to occur when divalent ions (like Copper and sulfate in this system) are present. Scaling is a serious issue that may limit the transport of ionic species from one compartment to the other, and thereby limiting performance of these systems. Prevention of this scaling is an important aspect of further study.

### Performance Comparison to Previous Studies

To improve MFC performance the cell design was improved compared to previous study (ter Heijne et al., 2010) by modifying the electrode materials, the membrane, and the distance between electrodes and mass transport within the system. **Table 1** compares the performance of the MFC described in this study to other studies that used copper reduction as cathode reaction. Main differences between this and other studies are: cell configuration, type of membrane, and different electrode materials. For example, all studies list in **Table 1** except Modin et al. (2012) and present study used carbon electrodes as cathode instead metal electrodes. The maximum current density in short circuit conditions produced by Modin et al. (2012) cell, was 7 A m^-2^ for 128 cm^2^ of anode felt surface using a small cathode surface of 1 cm^2,^, and using also an AEM. Even though, ter Heijne et al. (2010) achieved 3.2 A m^-2^ using graphite paper electrodes with a surface of 20 cm^2^ and a BPM. Cheng et al. (2013) achieved 1.2 A m^-2^ using a carbon fiber brush anode and a carbon rod cathode. On the other hand, this study achieved a current density of 23 A m^-2^ for 100 cm^2^ of anode graphite felt surface and 100 cm^2^ copper cathode and using AEM.

**Table 1:** Microbial fuel cell (MFC) comparison between different authors and cathode configurations.

Anode material and surface area	Cathode material and surface area	Membrane used	Voc (V)	Jpeak (A m^-2^)	Pmax (W m^-2^)	Reference
Graphite felt of 100 cm^2^	Copper plate of 100 cm^2^	AEM	0.485	23.0	5.50	This study
Graphite paper of 20 cm^2^	Graphite paper of 20 cm^2^	BPM	0.600	3.20	0.80	ter Heijne et al. (2010)
Carbon fiber brush	Carbon rod of 4.7 cm^2^	AEM	0.590	1.20	0.28	Cheng et al. (2013)
Graphite plate/graphite felt of 147 cm^2^	Graphite plate of 27 cm^2^	PEM	0.480	2.00^∗^	0.24^∗^	Tao et al. (2011)
2 × Graphite felt of 29.6 cm^2^	Titanium Wire of 1.02 cm^2^	AEM	-	7.00	-	Modin et al. (2012)
4 × Graphite felt of 22.5 cm^2^	Graphite plate of 22.5 cm^2^	AEM	0.550	0.90	0.20	Zhang et al. (2012)

^∗^The measurements of Tao et al. (2011) are expressed in A m^-3^ and W m^-3^ for current and power densities.

Although previous studies reported considerable current densities, the power production was not always equivalent due to, either short circuit conditions or high internal resistance. However, in this study the produced power density was 5.5 W m^-2^, which is considerably higher than the reported before with power densities ranging between 0.2 and 0.8 W m^-2^. This achievement is explained as a result of the low voltage losses of this MFC compared to other MFC configurations.

### Copper Removal and Analysis of the Deposited Copper

Samples from catholyte were taken and analyzed five times per week. Interestingly, during the first 100 h, we found out a slight increase in copper concentration from 2.0 g/L to 2.2 g/L. In this time period, the current density was still low, below 1 A m^-2^. Only when current density exceeds 1.0 A m^-2^, copper concentration started to drop and showed a steady decrease to 0.2 g/L after 24 h. A reasonable explanation for the increase in copper concentration may be that the copper electrode itself was oxidized to copper (II) under acidic conditions (pH 3). In order to check that copper oxidation occurs at acidic pH, a parallel experiment at open circuit in the same acidic conditions and copper concentration was performed. Here, a loss in weight of the electrode of 1.72 g of copper was observed after 24 h. This confirmed that the copper electrode can be oxidized under acidic conditions and explains the increase in copper concentration at low current densities (below 1 A m^-2^). Finally, it is important to mention that no copper was detected in the anode during any of the experiments.

After 1 month of operation, the cell was disassembled and the copper layer on the cathode was analyzed for purity. **Figures 5C,D** show the SEM images of the copper deposited on top of the copper cathode. Copper exhibited dendritic formations, which are likely a result of the high current densities. These dendrites crossed the spacer and touched the membrane surface. This is a point of attention because these structures might damage the membrane. Analysis of the Copper electrode by nitric acid leaching showed that the composition of the deposited copper on the cathode has a copper purity of 99.4 ± 1.0% with 0.3% in zinc. XRD analysis confirmed the copper purity of the copper deposited onto the electrode.

### Implications

High current density and power production were achieved in this improved cell design compared to the proof of principle by ter Heijne et al. (2010). The current was enhanced from 3.2 to 23 A m^-2^ while the power density increased from 0.8 to 5.5 W m^-2^.

State of the art technologies like solvent extraction and electrowinning SX/EW consume 2.716 kWh Kg^-1^ of Cu (Alvarado, 2002). On the contrary, this study produced a constant power of 3.7 W m^-2^ equivalent to an energy production of 0.081 kWh Kg^-1^ of Cu. It should be mentioned that, the energy required to operate the system was not taken into account here. Also, 2.056 kg of acetate is required per kilogram of copper, which can be considered as a waste product.

The current density can be improved even further by supplying an external voltage to the system. Although the produced current and removed amount of copper will be higher, this would go at the expense of energy input.

Analysis of the voltage losses showed that anode was the main contribution to the voltage losses in the system at low current densities, but when currents were higher the cathode and the membrane started to limit the system. Consequently, the transport of ionic species through the system and membrane should be studied in more detail, as well as the scaling on the membrane surface related to the ion transport are an important aspect that needs attention in the future. However, to improve the bioanode performance further, many strategies have been considered for future research like Inhibition of methanogens and sulfate reducers to improve coulombic efficiency. Recently also some studies had improved the bioanode by improving electrode material by organic and inorganic coatings on top, that improves the amount of bacteria that can be attached onto the electrode (Zhang et al., 2011, 2015).

Several challenges remain to be solved before this technology becomes practically applicable. The use of a copper electrode, which is normally used in electro-winning, may pose difficulties in MFCs. Those difficulties are typically the slow reaction rates and the fact that copper actually dissolves. Further research on copper deposition at low current densities with other metallic electrodes, such as stainless steel or titanium, would give more insights on this system performance.

## Conflict of Interest Statement

The authors declare that the research was conducted in the absence of any commercial or financial relationships that could be construed as a potential conflict of interest.

## References

[B1] Alvarado S. (2002). Long term energy-related environmental issues of copper production. *Energy* 27 183–196. 10.1016/S0360-5442(01)00067-6

[B2] Cheng S.-A. Wang B.-S. Wang Y.-H. (2013). Increasing efficiencies of microbial fuel cells for collaborative treatment of copper and organic wastewater by designing reactor and selecting operating parameters. *Bioresour. Technol.* 147 332–337. 10.1016/j.biortech.2013.08.040 23999262

[B3] Foley J. M. Rozendal R. A. Hertle C. K. Lant P. A. Rabaey K. (2010). *Life Cycle Assessment of High-Rate Anaerobic Treatment, Microbial Fuel Cells, and Microbial Electrolysis Cells.* Available at: http://pubs.acs.org.ezproxy.library.wur.nl/doi/full/10.1021/es100125h [accessed March 26, 2015]. 10.1021/es100125h 20356090

[B4] Geman H. Smith W. O. (2013). Theory of storage, inventory and volatility in the LME base metals. *Resour. Policy* 38 18–28. 10.1016/j.resourpol.2012.06.014

[B5] Hamilton W. A. (1985). Sulphate-reducing bacteria and anaerobic corrosion. *Annu. Rev. Microbiol.* 39 195–217. 10.1146/annurev.mi.39.100185.001211 3904600

[B6] Kuntke P. Sleutels T. Saakes M. Buisman C. J. N. (2014). Hydrogen production and ammonium recovery from urine by a Microbial Electrolysis Cell. *Int. J. Hydrogen Energy* 39 4771–4778. 10.1016/j.ijhydene.2013.10.089

[B7] Logan B. E. Call D. Cheng S. Hamelers H. V. M. Sleutels T. H. J. A. Jeremiasse A. W. (2008). Microbial electrolysis cells for high yield hydrogen gas production from organic matter. *Environ. Sci. Technol.* 42 8630–8640. 10.1021/es801553z 19192774

[B8] Modin O. Wang X. Wu X. Rauch S. Fedje K. K. (2012).Bioelectrochemical recovery of Cu, Pb, Cd, and Zn from dilute solutions. *J. Hazard. Mater.* 235–236, 291–297. 10.1016/j.jhazmat.2012.07.058 22910451

[B9] Muyzer G. Stams A. J. M. (2008). The ecology and biotechnology of sulphate-reducing bacteria. *Nat. Rev. Microbiol.* 6 441–454. 10.1038/nrmicro1892 18461075

[B10] Norgate T. E. Rankin W. J. (2002). *The Role of Metals in Sustainable Development, 49–55.* Available at: http://www.scopus.com/inward/record.url?eid=2-s2.0-2342565850&partnerID=40&md5=87a444cdfc8c05a408550a2a5b33804b

[B11] Pant D. Singh A. Van Bogaert G. Gallego Y. A. Diels L. Vanbroekhoven K. (2011). An introduction to the life cycle assessment (LCA) of bioelectrochemical systems (BES) for sustainable energy and product generation: relevance and key aspects. *Renew. Sustain. Energy Rev.* 15 1305–1313. 10.1016/j.rser.2010.10.005

[B12] Rozendal R. A. Hamelers H. V. M. Molenkamp R. J. Buisman C. J. N. (2007). Performance of single chamber biocatalyzed electrolysis with different types of ion exchange membranes. *Water Res.* 41 1984–1994. 10.1016/j.watres.2007.01.019 17343894

[B13] Sleutels T. H. Hamelers H. V. Buisman C. J. (2011). Effect of mass and charge transport speed and direction in porous anodes on microbial electrolysis cell performance. *Bioresour. Technol.* 102 399–403. 10.1016/j.biortech.2010.06.018 20619642

[B14] Sleutels T. H. J. A. Hamelers H. V. M. Rozendal R. A. Buisman C. J. N. (2009a). Ion transport resistance in Microbial Electrolysis Cells with anion and cation exchange membranes. *Int. J. Hydrogen Energy* 34 3612–3620. 10.1016/j.ijhydene.2009.03.004

[B15] Sleutels T. H. J. Lodder R. Hamelers H. V. M. Buisman C. J. N. (2009b). Improved performance of porous bio-anodes in microbial electrolysis cells by enhancing mass and charge transport. *Int. J. Hydrogen Energy* 34 9655–9661. 10.1016/j.ijhydene.2009.09.089

[B16] Sleutels T. H. J. A. Ter Heijne A. Buisman C. J. N. Hamelers H. V. M. (2012). Bioelectrochemical systems: an outlook for practical applications. *ChemSusChem* 5 1012–1019. 10.1002/cssc.201100732 22674691

[B17] Tao H. C. Liang M. Li W. Zhang L. J. Ni J. R. Wu W. M. (2011). Removal of copper from aqueous solution by electrodeposition in cathode chamber of microbial fuel cell. *J. Hazard. Mater.* 189 186–192. 10.1016/j.jhazmat.2011.02.018 21377788

[B18] ter Heijne A. Hamelers H. V. M. de Wilde V. Rozendal R. A. Buisman C. J. N. (2006). A bipolar membrane combined with ferric iron reduction as an efficient cathode system in microbial fuel cells. *Environ. Sci. Technol.* 40 5200–5205. 10.1021/es0608545 16999089

[B19] ter Heijne A. Hamelers H. V. M. Saakes M. Buisman C. J. N. (2008). Performance of non-porous graphite and titanium-based anodes in microbial fuel cells. *Electrochim. Acta* 53 5697–5703. 10.1016/j.electacta.2008.03.032

[B20] ter Heijne A. Liu F. Weijden R. V. Weijma J. Buisman C. J. Hamelers H. V. (2010). Copper recovery combined with electricity production in a microbial fuel cell. *Environ. Sci. Technol.* 44 4376–4381. 10.1021/es100526g 20462261

[B21] Vegliò F. Quaresima R. Fornari P. Ubaldini S. (2003). Recovery of valuable metals from electronic and galvanic industrial wastes by leaching and electrowinning. *Waste Manag.* 23 245–252. 10.1016/S0956-053X(02)00157-5 12737966

[B22] Watkins C. McAleer M. J. (2006). Pricing of non-ferrous metals futures on the London Metal Exchange. *Appl. Finan. Econ.* 16 853–880. 10.1080/09603100600756514

[B23] Zhang C. Liang P. Jiang Y. Huang X. (2015). Enhanced power generation of microbial fuel cell using manganese dioxide-coated anode in flow-through mode. *J. Power Sources* 273 580–583. 10.1016/j.jpowsour.2014.09.129

[B24] Zhang L. J. Tao H. C. Wei X. Y. Lei T. Li J. B. Wang A. J. (2012). Bioelectrochemical recovery of ammonia-copper(II) complexes from wastewater using a dual chamber microbial fuel cell. *Chemosphere* 89 1177–1182. 10.1016/j.chemosphere.2012.08.011 22944254

[B25] Zhang Y. Hu Y. Li S. Sun J. Hou B. (2011). Manganese dioxide-coated carbon nanotubes as an improved cathodic catalyst for oxygen reduction in a microbial fuel cell. *J. Power Sources* 196 9284–9289. 10.1016/j.jpowsour.2011.07.069

